# Do Health Inequities Increase Risk For Healthcare Associated Infections In Acute Care Settings?

**DOI:** 10.1017/ash.2025.204

**Published:** 2025-09-24

**Authors:** Michael Vollmer, Cristine Lacerna, Kara Mullane, Frederick Cabasa

**Affiliations:** 1 The Permanente Medical Group; 2 Kaiser Permanente; 3 Kaiser Permanente; 4 TPMG Kaiser Permanente

## Abstract

**Background:** The COVID-19 pandemic highlighted health inequities with rates of illness and outcomes among various populations. This project evaluates factors involved with health disparities in patients with identified hospital-associated infections (HAIs). Identifying and targeting these inequities as risk factors could reduce HAIs in affected groups. **Method:** We examined HAIs reported to National Health and Safety Network (NHSN) from a large integrated health network, including 21 acute care hospitals in Northern California. This data set included Methicillin-resistant Staphylococcus aureus (MRSA), Clostridioides difficile infection (CDI), and Vancomycin-resistant enterococci (VRE) infections, catheter-associated urinary tract infections (CAUTI), central line associated bloodstream infections (CLABSI), and surgical site infections (SSI) from 29 procedures. The analysis included 6,813 reported cases of HAI from 2019 to 2023. Data was stratified with equity, inclusion, and diversity risk factors, and employing multivariate regression analysis to calculate odds ratios for infection. **Result:** Spanish-speaking patients had increased odds ratios for CLABSI (1.8, p=0.003), CAUTI (2.08, p=<0.0001). **Conclusions:** The study identifies those with Spanish as preferred language, using interpreters, or family or friends as interpreters, as all having a higher risk for acquiring an HAI. These differences remain after accounting for known risk factors of age, gender, body max index (BMI), length of stay, emergency admissions, and comorbidity risk. This suggest that including and analyzing health inequity risk factors may help in early intervention to reduce or prevent HAIs.

